# Skin Autofluorescence Measurement as Initial Assessment of Hepatic Parenchyma Quality in Patients Undergoing Liver Resection

**DOI:** 10.3390/jcm11185341

**Published:** 2022-09-11

**Authors:** Maciej Krasnodębski, Marcin Morawski, Jan Borkowski, Karolina Grąt, Jan Stypułkowski, Michał Skalski, Andriy Zhylko, Marek Krawczyk, Michał Grąt

**Affiliations:** 1 Department of General, Transplant, and Liver Surgery, Medical University of Warsaw, 02-097 Warsaw, Poland; 2 Second Department of Clinical Radiology, Medical University of Warsaw, 02-097 Warsaw, Poland

**Keywords:** liver steatosis, liver fibrosis, liver resection, skin autofluorescence

## Abstract

Skin autofluorescence (SAF) can detect advanced glycation end products (AGEs) that accumulate in tissues over time. AGEs reflect patients’ general health, and their pathological accumulation has been associated with various diseases. This study aimed to determine whether its measurements can correlate with the liver parenchyma quality. This prospective study included 186 patients who underwent liver resections. Liver fibrosis and/or steatosis > 10% were found in almost 30% of the patients. ROC analysis for SAF revealed the optimal cutoff point of 2.4 AU as an independent predictor for macrovesicular steatosis ≥ 10% with an AUC of 0.629 (95% CI 0.538–0.721, *p* = 0.006), 59.9% sensitivity, 62.4% specificity, and positive (PPV) and negative (NPV) predictive values of 45.7% and 74.1%, respectively. The optimal cutoff point for liver fibrosis was 2.3 AU with an AUC of 0.613 (95% CI 0.519–0.708, *p* = 0.018), 67.3% sensitivity, 55.2% specificity, and PPV and NPV of 37.1% and 81.2%, respectively. In the multivariable logistic regression model, SAF ≥ 2.4 AU (OR 2.16; 95% CI 1.05–4.43; *p* = 0.036) and BMI (OR 1.21; 95% CI 1.10–1.33, *p* < 0.001) were independent predictors of macrovesicular steatosis ≥ 10%. SAF may enhance the available non-invasive methods of detecting hepatic steatosis and fibrosis in patients prior to liver resection.

## 1. Introduction

Advanced glycation end products (AGEs) are a heterogeneous group of glycated proteins, lipids, and nucleic acids created during the non-enzymatic glycation process. Their accumulation increases with age, but this process can be accelerated by diabetes, an unhealthy lifestyle, oxidative stress, or diseases with inflammatory processes [1,2,3]. Some AGE molecules can promote inflammatory mediators, such as cytokines, which makes them not only the consequence of a particular disease but also its stimulant [4]. AGEs can activate signaling pathways via specialized receptors. For example, the receptor for AGE (RAGE)-type receptor mechanism is responsible for inducing the production of reactive oxygen species, leading to cytotoxicity [5]. The natural reduction in AGE levels in the human body involves both enzymatic degradation and secretion through the kidneys [6]. One of the most well-known AGEs is glycated hemoglobin (HbA1c), which is the worldwide gold standard for glucose monitoring in diabetic patients [7]. AGEs can be classified in various ways, including as endogenous or exogenous (dietary) depending on their origin, as toxic or non-toxic, based on the precursor (such as glucose-, fructose-, or glyoxal-derived), by molecular weight, or as fluorescent or nonfluorescent [3].

Autofluorescence is the process of light re-emission by endogenous compounds when excited at a particular wavelength. This phenomenon can be used to indirectly measure substances that accumulate in the human skin. Skin autofluorescence (SAF) devices can detect compounds such as AGEs that accumulate over time in tissues [8,9,10]. Interestingly, SAF measurements resemble not only fluorescent AGEs but also other spectra of nonfluorescent glycation end products [11]. Moreover, SAF results correlate with other invasive AGE detection methods such as high-performance liquid chromatography [11,12].

In recent years, SAF has been mainly studied in diabetes and cardiovascular and renal diseases [1,13,14]. For instance, in diabetic patients, increased AGE levels were correlated with the severity of micro- and macrovascular complications, retinopathy, nephropathy, and atherosclerosis [15]. However, the utility of the SAF extends beyond these diseases. For instance, AGE accumulation in the skin was studied by our group in patients undergoing liver resection, and its higher levels were linked to acute kidney injury in the postoperative period. Importantly, the association between SAF and kidney injury was also independent of diabetes or body mass index (BMI) [16].

Liver resection remains the core treatment for the majority of non-oncological and oncological diseases of that organ. The status of the liver parenchyma not only determines late outcomes but can also influence decision making when planning resection. Therefore, the anticipated future liver remnant volume after resection should be higher in patients with liver injuries such as steatohepatitis or cirrhosis [17].

To precisely determine the degree of liver parenchyma quality preoperatively, a percutaneous biopsy must be performed. Although this invasive procedure is safe in most cases, it can lead to distinct complications. On the other hand, in recent years, non-invasive tests have emerged that can compete with liver biopsies, such as ultrasound or magnetic resonance elastography (MRE), as well as novel biomarkers such as measurements of endogenously produced volatile organic compounds (VOCs) in exhaled breath [18,19,20]. In addition, simple blood biomarker tests such as the AST-platelet ratio index (APRI) or fibrosis score 4 can also be applied [21]. Likewise, the SAF is a non-invasive examination, and its results can represent the “overall” health status of the patients, thus potentially combining comorbidities and liver injury status. However, to date, only two preliminary reports have addressed its role in patients with cirrhosis [22,23]. Therefore, following our previous studies on the clinical utility of SAF, we aimed to determine whether its measurements can correlate with the quality of the liver parenchyma.

## 2. Materials and Methods

This prospective study included 186 patients treated in the Department of General, Transplant, and Liver Surgery at the Medical University of Warsaw between September 2018 and October 2020. The study cohort comprised patients who underwent liver resection. SAF measurements were assessed preoperatively using an AGE Reader device (Diagnostics Technologies B.V., Groningen, The Netherlands) based on photodiodes. The detection method begins with the application of the reader to the skin (the anterior side of the forearm). Ultraviolet radiation of wavelength with peak intensity at approximately 350–370 nm interacts with AGEs in the skin, and the light emitted and reflected is then assessed. The measurements were expressed in arbitrary units (AU). This process was repeated two more times to confirm the accuracy of the measurements.

All resected liver fragments were examined by local pathologists, and the degrees of liver fibrosis and steatosis were determined. Macrovesicular steatosis of the liver was used as the primary outcome measure. The ≥10% cutoff point was selected as follows: first, pathologists described the nonsignificant degree of steatosis in those with <5%; second, there were no values of steatosis reported between 5% and 10% in the final pathological examinations; and finally, the same cutoff point was selected in other studies on liver resections and steatosis [24,25,26]. Any stage of liver fibrosis (stage 1–4 according to the Batts–Ludwig system) was set as a secondary outcome measure [27]. Patients with missing SAF measurements or pathological examination of nontumoral liver tissue were excluded.

Quantitative and qualitative variables are presented as medians with interquartile ranges or numbers with a percentage of the total. The chi-square test and Mann–Whitney U test were used to compare subgroups. Receiver operating characteristic (ROC) analyses were performed to choose the optimal cutoff values for SAF in the prediction of fibrosis and macrovesicular steatosis. Odds ratios (ORs) and areas under the curve (AUCs) were presented with 95% confidence intervals (CIs). Logistic regression was used for univariate and multivariate analyses of predictors of liver fibrosis and steatosis. SAF measurements and variables related to patients’ overall health status were included in the model. Multivariable analysis was performed based on the stepwise method, using *p* < 0.05, for both the inclusion and exclusion of variables from the model. Spearman’s correlation coefficient was used for unadjusted analyses of the associations between SAF and patients’ age and BMI. The level of significance was set at *p* < 0.05. Statistical analyses were performed using STATISTICA v13 software (TIBCO Software Inc., Palo Alto, CA, USA).

## 3. Results

Liver fibrosis and/or steatosis > 10% were found in almost 30% of the patients. The main indication for liver resection was colorectal liver metastasis (43.6%). Baseline characteristics of the study cohort are presented in Table 1. A detailed description of the patients with cirrhosis is provided in Table 2.

**Figure 1:**
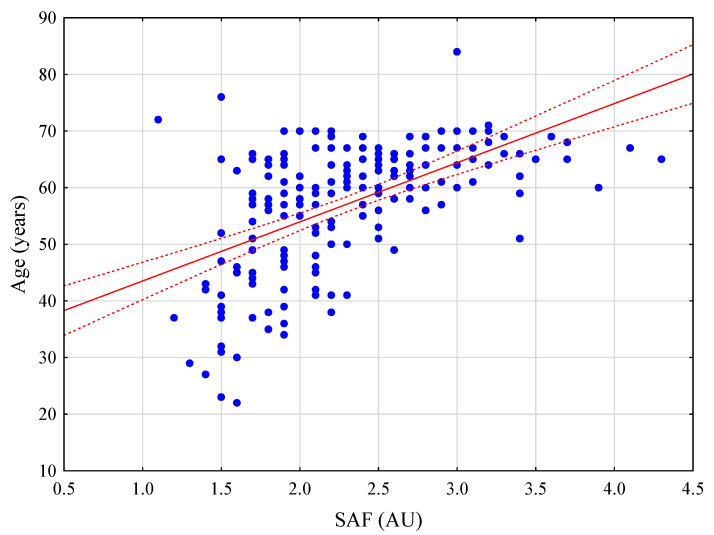
Correlation between skin autofluorescence (SAF) and age.

**Figure 2:**
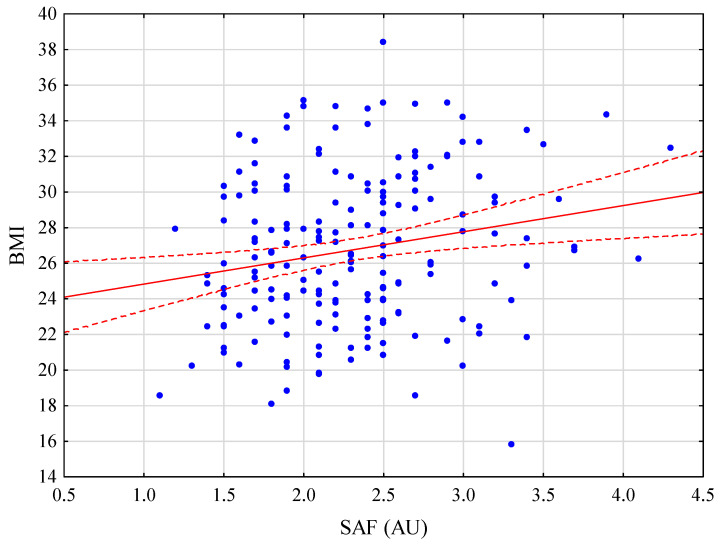
Correlation between skin autofluorescence (SAF) and body mass index (BMI).

**Figure 3:**
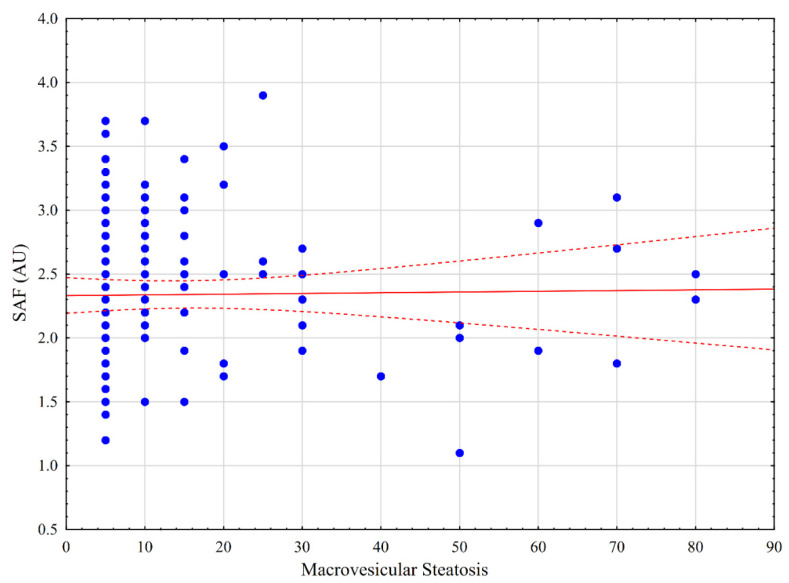
Correlation between skin autofluorescence (SAF) and degree of liver steatosis.

The SAF levels significantly differed between healthy patients and those with diabetes (*p* < 0.001), arterial hypertension (*p* < 0.001), BMI > 30 kg/m^2^ (*p* = 0.047), liver fibrosis (*p* = 0.020), and macrovesicular steatosis ≥ 10% (*p* = 0.008), as well as smokers (*p* < 0.001) and older patients (>65 years) (*p* < 0.001). No difference was observed in SAF levels between patients with and without chemotherapy (*p* = 0.746), serum bilirubin concentration ≥ 1.2 mg/dL (*p* = 0.990), and hepatitis C or B virus (HCV/HBV) (*p* = 0.199), as well as male and female patients (*p* = 0.107) and cirrhotic patients versus stage 1–3 liver fibrosis (*p* = 0.061, Table 3). SAF was positively correlated with patient age and BMI (R = 0.551, *p* < 0.001; R = 0.201, *p* = 0.006, respectively). Correlation charts are presented in Figure 1 and Figure 2. Among patients with any degree of steatosis and those with >30% macrovesicular steatosis, there was no difference between any stage of steatosis (*p* = 0.293). There was no correlation between the SAF and any stage of steatosis (Figure 3; R = 0.017; *p* = 0.858).

**Figure 4:**
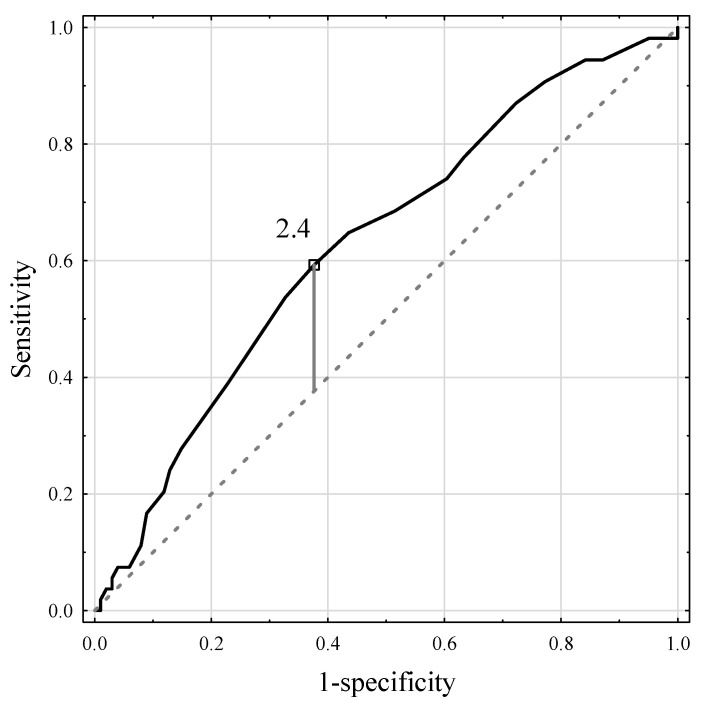
The ROC curve for the skin autofluorescence measurements and macrovesicular steatosis > 10%.

ROC analysis for SAF revealed the optimal cutoff point of 2.4 AU as an independent predictor for macrovesicular steatosis ≥ 10% with an AUC of 0.629 (95% CI 0.538–0.721, *p* = 0.006), 59.9% sensitivity, 62.4% specificity, and positive (PPV) and negative (NPV) predictive values of 45.7% and 74.1%, respectively (Figure 4). In the group of patients with SAF ≥ 2.4 AU, 32 (45.7%) patients had macrovesicular steatosis in ≥10% of the liver as compared to 22 (25.9%) patients in the SAF < 2.4 AU group (*p* = 0.010). 

The variable based on the optimal cutoff point of SAF ≥ 2.4 AU was included in the logistic regression model. In the univariate model, apart from SAF ≥ 2.4 AU (*p* = 0.011), other risk factors were diabetes (*p* = 0.034) and BMI (*p* < 0.001). In the multivariable logistic regression model, SAF ≥ 2.4 AU (OR 2.16; 95% CI 1.05–4.43; *p* = 0.036) and BMI (OR 1.21; 95% CI 1.10–1.33, *p* < 0.001, Table 4) were independent predictors of macrovesicular steatosis ≥ 10%.

**Figure 5:**
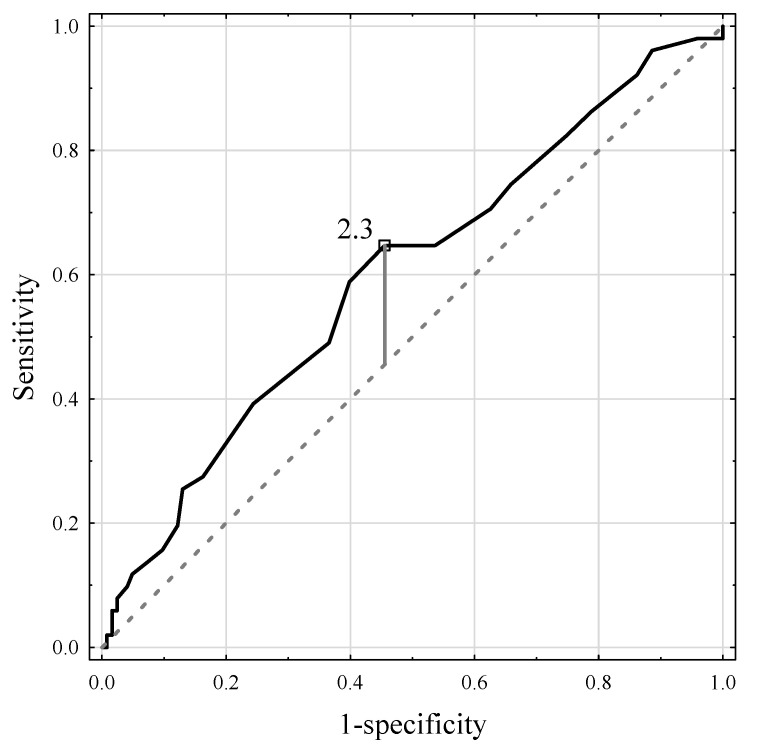
The ROC curve for the skin autofluorescence measurements and liver fibrosis.

The optimal cutoff point for liver fibrosis was 2.3 AU with an AUC of 0.613 (95% CI 0.519–0.708, *p* = 0.018), 67.3% sensitivity, 55.2% specificity, and PPV and NPV of 37.1% and 81.2%, respectively (Figure 5). In the group of patients with SAF ≥ 2.3 AU, 33 (37.1%) patients had liver fibrosis as compared to 16 (18.8%) patients in the SAF < 2.3 AU group (*p* = 0.009).

In the univariable logistic regression model, older age (OR 1.07; 95% CI 1.03–1.11; *p* = 0.001), SAF ≥ 2.3 AU (OR 2.54; 95% CI 1.13–5.08; *p* = 0.008), diabetes (OR 2.95; 95% CI 1.14–7.62, *p* = 0.026), smoking (OR 2.05; 95% CI 1.03–4.07, *p* = 0.040), HBV (OR 3.29; 95% CI 1.19–9.11, *p* = 0.022), and HCV (OR 17.30; 95% CI 2.03–147.84, *p* = 0.009) were significant risk factors for fibrosis. In the multivariate model, the independent predictors were older age (OR 1.06; 95% CI 1.02–1.11, *p* = 0.005), HBV (OR 2.99; 95% CI 1.02–8.76, *p* = 0.046), and HCV (OR 13.66; 95% CI 1.55–120.62, *p* = 0.019). Following the exclusion of patients with HCV and HBV in the multivariate model, the sole independent risk factor was patient age (Table 5).

## 4. Discussion

SAF is a new biomarker for the detection of AGEs, the accumulation of which has been linked to various diseases. Our study showed that it can also be used to assess the health of the liver. SAF measurements in patients undergoing liver resection can be used as a screening test to assess the condition of the liver before surgery because of the high NPV in patients with fatty liver and fibrosis. Importantly, the elevated SAF measurements in our study in elderly and diabetic patients are consistent with those in previous studies [15,28,29]. Similarly, a higher SAF was associated with an increase in BMI [30]. 

In our analysis, increased levels of SAF were found in patients with liver steatosis, as well as in those with liver injury and fibrosis. Similarly, AGEs detected in serum were found to be elevated in liver steatosis and cirrhosis in various studies [31,32,33,34,35]. AGEs can promote oxidative stress and cellular dysfunction in the liver, leading to steatosis and non-alcoholic fatty liver disease (NAFLD). AGE metabolism occurs mainly in sinusoidal endothelial cells and Kupfer liver cells [36,37]. This process can create a vicious circle; on the one hand, AGEs worsen hepatic metabolism, and these impaired catabolic processes lead to an even greater accumulation of glycation end products [38]. The key role in liver detoxification was further confirmed when the levels of plasma AGEs (fluorescent AGEs and Nε-carboxymethyllysine) were significantly lower after liver transplantation [33]. 

In contrast to measurements of serum AGEs using spectrofluorimetry and enzyme-linked immunosorbent assay (ELISA), SAF examination to assess AGEs and liver status has not been well-determined [3]. To the best of our knowledge, no study has addressed SAF and liver steatosis. There have been no studies on liver fibrosis and SAF; however, two short reports by Maury et al. and Rye et al. addressed the role of SAF in patients with cirrhosis [22,23]. In their pilot study, Maury et al. tested the SAF in 32 patients with cirrhosis, 16 patients with type 2 diabetes, and 7 healthy control subjects. The results for the three groups were a mean SAF of 2.4, 3.4, and 1.9 AU, respectively. Interestingly, in cirrhotic patients with icterus, the mean SAF was lower (2.2 AU) than that in cirrhotic patients without icterus (2.7 AU). This result was explained by the utility of bilirubin in absorbing particular wavelengths, thus potentially lowering the measurements [22]. Similarly, skin pigmentation can affect AGE readers, as has been previously reported [39,40]. In our study, the potential effect of skin pigmentation was dismissed by a homogenous cohort of patients falling into the Fitzpatrick scale class between I and III [41]. Cirrhotic patients in our study, apart from one patient, had serum bilirubin concentrations of <1.2 mg/dL. However, the SAF measurements in our analysis for that particular group had a lower median than those of patients with less severe fibrosis or those with diabetes. This phenomenon cannot be easily explained, and the low number of patients with cirrhosis in our study (n = 7) can be considered for some potential confounding effects of the small sample size analysis. On the other hand, in the report by Rye et al., cirrhotic patients had a mean SAF that was not significantly different from the non-cirrhotic control group when patients with diabetes and ischemic heart disease were excluded. Initially, in this analysis, there were 28 patients with cirrhosis; however, as the full-text version was not available and the study was only presented in abstract form, it was not possible to determine how many patients were finally analyzed [23]. The question that can be raised is whether cirrhotic patients have a higher accumulation of AGEs that are not fluorescent; therefore, their accumulation in the skin of cirrhotic patients disrupts the skin autofluorescence that is detected through SAF or other substances, as previously described for bilirubin [22]. Overall, this surprising outcome cannot be explained based on our data.

Apart from fibrosis, our analysis revealed that patients with SAF ≥ 2.4 AU bear a two-fold risk for macrovesicular steatosis diagnosis. The clinical application of these results can be used to stratify patient risk when combined with other clinical data and diagnostics. The SAF examination has a high NPV and can be used with other diagnostics to rule out potential underlying liver disease without invasive tests in clinical settings. The incorporation of patients’ overall health status with the degree of liver damage in non-invasive SAF measurements opens a new potential method to stratify the risk for future surgical patients. As opposed to other diagnostic methods such as ultrasound computed tomography or magnetic resonance imaging, SAF measurements are easily accessible, and outcomes are expressed in numbers. MRE and ultrasound elastography are modern non-invasive methods to determine the degree of fibrosis; however, they are expensive, require trained specialists, and are more time-consuming than SAF. However, the low specificity of SAF in detecting patients at risk of fibrosis and steatosis probably rules out the possibility of using it as a sole diagnostic tool. To increase specificity, the combination of SAF and APRI or FIB4 scores could be applied in the future to detect or monitor liver fibrosis and patients with NAFLD [42]. 

To address the limitations of this study, first, there is a great abundance of different AGEs, both fluorescent and nonfluorescent. The outcome of SAF cannot be used to determine the specific AGE subgroups. Although skin biopsies revealed a correlation between autofluorescent and nonfluorescent AGE, there is a great chance of a distinct group of nondetectable AGEs that can affect patients [11]. Second, the correlation of age and BMI with SAF was confirmed in this study; however, no adjusted outcome of SAF was available according to these variables. Regarding other possible confounding factors, while data on smoking, diabetes, and hypertension were included in the analysis, there were no data on ischemic heart disease in the cohort. Third, SAF was not compared to other non-invasive measurements (i.e., VOC) to determine liver fibrosis. Moreover, the plasma levels of AGEs, endothelial cells, and Kupffer cells, which are prominent in the metabolism of AGEs, were not investigated in this analysis. Finally, the risk score based on BMI and age with comorbidities could have increased sensitivity and specificity in assessing liver injury; however, a well-selected healthy control group can overcome this limitation. 

## 5. Conclusions

SAF is moderately associated with liver steatosis but not with its degree or fibrosis. In our opinion, SAF can be used as a preliminary method to select patients for more ac-curate diagnostic methods or as an additional method to other non-invasive tests to enhance their precision. However, this requires further study for confirmation.

## Figures and Tables

**Table 1:** Baseline characteristics.

	Median (IQR) or n (%)
Patient sex	
male	91 (48.9%)
female	95 (51.1%)
Patient age (years)	60 (51–65)
SAF (AU)	2.3 (1.9–2.6)
Body mass index (kg/m^2^)	26.4 (23.5–30.1)
HBV	18 (9.7%)
HCV	7 (3.8%)
Chemotherapy	86 (46.2%)
Diabetes	24 (12.9%)
Arterial hypertension	63 (33.9%)
Smokers	84/181 (46.4%)
Cirrhosis	7 (3.8%)
Liver fibrosis	49/174 (28.2%)
Macrovesicular steatosis ≥ 10%	54/155 (34.8%)
Macrovesicular steatosis ≥ 20%	26/155 (16.8%)
Macrovesicular steatosis ≥ 30%	18/155 (11.6%)
Preoperative laboratory tests	
white blood count (10^3^/mm^3^)	6.3 (5.3–7.8)
hemoglobin (g/dL)	13.5 (12.6–14.2)
platelets (10^3^/mm^3^)	222 (181–263)
creatinine (mg/dL)	0.8 (0.7–0.9)
bilirubin (mg/dL)	0.5 (0.4–0.7)
INR	1.0 (1.0–1.1)
albumin (g/dL)	4.4 (4.1–4.6)
AST (U/L)	28 (23–39)
ALT (U/L)	27 (20–41)
Primary diagnosis	
colorectal liver metastases	81 (43.6%)
hepatocellular cancer	22 (11.8%)
gallbladder cancer	17 (9.1%)
cholangiocarcinoma	19 (10.2%)
other	47 (25.3%)

IQR, interquartile range; SAF, skin autofluorescence; AU, arbitrary unit; INR, international normalized ratio; HBV, hepatitis B virus; HCV, hepatitis C virus; AST, aspartate aminotransferase; ALT, alanine aminotransferase.

**Table 2:** Description of cirrhotic patients.

Patient	Age	Cause of Cirrhosis	Indication to Surgery	Child-Pugh Class	Albumin (g/dL)	Bilirubin (mg/dL)	INR	Ascites	Encephalopathy	PLT (10²/µL)	Portal Hypertension	SAF (AU)
1. Female	63	HCV *	HCC	A	5.0	0.68	1.08	absent	-	126	-	1.6
2. Male	45	HBV	HCC	A	4.9	0.39	1.29	absent	-	247	-	2.1
3. Male	62	Alcohol	HCC	A	4.9	0.28	1.04	absent	-	157	-	2.6
4. Male	65	HCV *	HCC	A	4.5	0.31	1.08	absent	-	250	-	2.5
5. Female	66	HCV	HCC	A	4.4	2.35	1.23	absent	-	95	-	1.7
6. Male	76	HCV *	HCC	A	4.3	0.43	1.07	absent	-	121	yes	1.5
7. Male	55	Alcohol	HCC	A	3.8	0.61	1.11	absent	-	167	-	2.4

HCC, hepatocellular carcinoma; HCV, hepatitis C virus; HBV, hepatitis B virus; * after eradication.

**Table 3:** Distribution of skin autofluorescence (SAF) among patient variables.

Variable	Median (AU)		Median (AU)	*p*
Male	2.4	Female	2.2	0.107
Age ≥ 65 years	2.6	Age < 65 years	2.1	<0.001
BMI ≥ 30	2.4	BMI < 30	2.2	0.047
Diabetes	2.7	No diabetes	2.2	<0.001
Smoking	2.5	Non-smokers	2.1	<0.001
Chemotherapy	2.3	No chemotherapy	2.2	0.746
Arterial hypertension	2.5	No arterial hypertension	2.1	<0.001
Liver fibrosis	2.5	No fibrosis	2.2	0.020
Cirrhosis	2.1	Liver fibrosis (Stage 1–3)	2.5	0.061
Serum bilirubin ≥ 1.2 mg/dL	2.2	Serum bilirubin < 1.2 mg/dL	2.3	0.990
INR ≥ 1.2	2.1	INR < 1.2	2.3	0.727
Albumin ≤ 3.5 g/dL	2.2	Albumin > 3.5 g/dL	2.4	0.182
AST ≥ 40 (U/L)	2.2	AST < 40 (U/L)	2.4	0.462
ALT ≥ 40 (U/L)	2.3	ALT < 40 (U/L)	2.2	0.523
Macrovesicular steatosis ≥ 10%	2.5	Macrovesicular steatosis < 10%	2.2	0.008
HBV or HBV infection	2.5	No viral infection	2.2	0.199

AU—arbitrary units; BMI—body mass index; HBV—hepatitis B virus; HCV—hepatitis C virus; INR—international normalized ratio; AST—aspartate aminotransferase; ALT—alanine aminotransferase.

**Table 4:** Logistic regression model for macrovesicular steatosis ≥ 10%.

	OR (95% CI)	*p*	OR (95% CI)	*p*
Age	1.03 (0.99–1.07)	0.055	-	-
BMI	1.22 (1.11–1.34)	<0.001	1.21 (1.10–1.33)	<0.001
SAF ≥ 2.4	2.41 (1.23–4.74)	0.011	2.16 (1.05–4.43)	0.036
Diabetes	3.05 (1.09–8.55)	0.034	-	-
Chemotherapy	0.94 (0.49–1.83)	0.860	-	-
Smoking	1.72 (0.88–3.83)	0.115	-	-

OR, odds ratio; 95% CI, 95% confidence interval; BMI, body mass index; SAF, skin autofluorescence.

**Table 5:** Logistic regression model for liver fibrosis.

	OR (95% CI)	*p*	OR (95% CI)	*p*
Age	1.07 (1.03–1.11)	0.001	1.06 (1.02–1.11)	0.005
BMI	1.04 (0.96–1.12)	0.333	-	-
SAF ≥ 2.3	2.54 (1.13–5.08)	0.008	-	-
Diabetes	2.95 (1.14–7.62)	0.026	-	-
Smoking	2.05 (1.03–4.07)	0.040	-	-
HCV	17.30 (2.03–147.84)	0.009	13.66 (1.55–120.62)	0.019 *
HBV	3.29 (1.19–9.11)	0.022	2.99 (1.02–8.76)	0.046 *

OR—odds ratio; 95% CI—95% confidence interval; BMI—body mass index; SAF—skin autofluorescence, HCV—hepatitis C virus; HBV—hepatitis B virus; * after excluding viral hepatitis in multivariate model, the sole independent risk factor was age.

## Data Availability

The datasets generated during and/or analyzed during the current study are available from the corresponding author on reasonable request.
